# Inversion 2La is associated with enhanced desiccation resistance in *Anopheles gambiae*

**DOI:** 10.1186/1475-2875-8-215

**Published:** 2009-09-21

**Authors:** Emilie M Gray, Kyle AC Rocca, Carlo Costantini, Nora J Besansky

**Affiliations:** 1 Eck Institute for Global Health, Department of Biological Sciences, University of Notre Dame, Notre Dame, IN, USA; 2 Institut de Recherche pour le Développement (IRD), UR016, and Laboratoire de Recherche sur le Paludisme, Organisation de Coordination pour la lutte contre les Endémies en Afrique Centrale (OCEAC), Yaoundé, Cameroon

## Abstract

**Background:**

*Anopheles gambiae*, the principal vector of malignant malaria in Africa, occupies a wide range of habitats. Environmental flexibility may be conferred by a number of chromosomal inversions non-randomly associated with aridity, including 2La. The purpose of this study was to determine the physiological mechanisms associated with the 2La inversion that may result in the preferential survival of its carriers in hygrically-stressful environments.

**Methods:**

Two homokaryotypic populations of *A. gambiae *(inverted 2La and standard 2L+^a^) were created from a parental laboratory colony polymorphic for 2La and standard for all other known inversions. Desiccation resistance, water, energy and dry mass of adult females of both populations were compared at several ages and following acclimation to a more arid environment.

**Results:**

Females carrying 2La were significantly more resistant to desiccation than 2L+^a ^females at emergence and four days post-emergence, for different reasons. Teneral 2La females had lower rates of water loss than their 2L+^a ^counterparts, while at four days, 2La females had higher initial water content. No differences in desiccation resistance were found at eight days, with or without acclimation. However, acclimation resulted in both populations significantly reducing their rates of water loss and increasing their desiccation resistance. Acclimation had contrasting effects on the body characteristics of the two populations: 2La females boosted their glycogen stores and decreased lipids, whereas 2La females did the contrary.

**Conclusion:**

Variation in rates of water loss and response to acclimation are associated with alternative arrangements of the 2La inversion. Understanding the mechanisms underlying these traits will help explain how inversion polymorphisms permit exploitation of a heterogeneous environment by this disease vector.

## Background

In tropical environments, water availability can be a major factor limiting insect distribution [1]. As such, the geographic and seasonal range of the mosquito *Anopheles gambiae*, the principal African vector of malaria, depends on its ability to survive in arid environments [2]. Its small size results in a large surface area to volume ratio over which evaporative water loss can occur. The activity of adult mosquitoes exacerbates the risk of water loss, yet the scale of malaria morbidity and mortality attest to the mosquito's success throughout most of the continent [3].

It is thought that *A. gambiae *owes its broad distribution to a number of polymorphic chromosomal inversions within its genome, which are proposed to confer a diverse array of adaptations to the species as a whole. The patterns of inversion polymorphism shift both geographically and seasonally. Inversion frequencies have been associated with factors such as aridity [4,5], larval habitat preference [6,7], *Plasmodium *infection rates [8] and insecticide resistance [9]. In particular, some inversions are so strongly associated with climatic factors that climate-based models can be used to predict the presence/absence of these inversions with a high degree of accuracy [10]. However, the physiological mechanisms by which these inversions confer an adaptive advantage in a given environment remain unknown.

One inversion particularly strongly linked to aridity clines in West and Central Africa is 2La. The 2La inversion is absent in the southern parts of Nigeria and Cameroon and progressively increases in frequency, reaching fixation in the arid north of these countries [5,11]. It is hypothesized that this inversion has captured a set of alleles, which together confer an advantage to its carriers in arid conditions [4,12]. Accordingly, the 2La inversion may facilitate *A. gambiae *survival and consequent malaria transmission in the more xeric parts of its distribution, and may have contributed to the range expansion of this disease vector from the forest fringe into the savanna [13].

Dehydration stress, a particular threat to small arthropods in arid environments, can be limited by one of three mechanisms: increasing body water stores, reducing the rate at which water is lost, or increasing the tolerance to water loss. By modifying any of these traits, a mosquito can increase its resistance to desiccation (DR). In *Drosophila melanogaster*, various traits may evolve in response to selection for enhanced DR [see [14] and references therein]. However, a common finding is that water loss rates tend to be lower in xeric species than their mesic counterparts [15], an extreme example of which is seen among desert beetles [16]. Water loss rate is also known to vary in *Culex pipiens *mosquitoes, which drastically reduce their rates of water loss during diapause [17].

Recent studies have revealed variation in DR among *Anopheles. Anopheles arabiensis*, a sibling species to *A. gambiae*, inhabits the more arid parts of the *A. gambiae *species complex range in tropical Africa, and is more resistant to desiccation than *A. gambiae s.s*. [18]. This difference was shown to be due to higher body water stores in *A. arabiensis*, but any connection between this trait and the chromosomal inversions that distinguish both species has not been explored. Within *A. gambiae s.s*., variation in DR has been associated with molecular forms in Mali named M and S [19], but the physiological mechanisms responsible for this variation and their relationship to karyotype differences between forms have not been investigated. Although these studies suggest the presence of genetic variation for DR among members of the *A. gambiae *species complex, the contribution of individual inversions to this phenotype has yet to be examined.

The current study explores variation in DR that is specifically associated with the 2La inversion of *A. gambiae s.s*. and may explain its differential distribution in the field. To isolate the effect of this inversion on the mosquito's physiology, the laboratory strain of *A. gambiae *was polymorphic solely for the 2La inversion, but fixed and standard for all other inversions. A recent and complementary study by White *et al *[12] adopted a genotypic approach to identify two regions of high genetic differentiation between alternative arrangements of 2La, encompassing >200 genes within the inversion. The present phenotypic approach may help implicate candidate genes within the inversion that contribute to enhanced aridity tolerance, while shedding light on the mechanisms allowing *A. gambiae *to exploit environmental heterogeneities and adapt to a changing climate.

## Methods

### Colonies

Two homokaryotypic sub-strains of *A. gambiae *used in this study were created from a parental strain that is polymorphic for 2La but fixed and standard for all other inversions. The parental strain (SUCAM) originated early in 2005 from a cross between CAM (2R+/2L+^a^) and SUA (2R+/2La) colonies, both representing the M molecular form of *A. gambiae *derived from regions near Yaoundé, Cameroon and Suakoko, Liberia, respectively. After approximately 36 generations of intermating within SUCAM (assuming one generation per month), homokaryotypic sub-strains, named SUCAM 2La and SUCAM 2L+^a^, were created in April 2008 by identifying the 2L karyotype of live adults using DNA from one leg. On the morning of adult emergence, mosquitoes were cold anesthetized, amputated of one rear leg and isolated in a numbered vial. Each leg was ground in lysis buffer [20] and PCR was performed using 2La and 2L+^a ^specific primers [21]. By early afternoon, the 2L karyotype of each mosquito was known, allowing placement of each mosquito in the appropriate cage. Selection was terminated when each population cage contained at least 70 individuals. Experiments were initiated after three generations. All mosquito populations were maintained at 27°C and 80% RH, except where otherwise noted. Mosquitoes tested at emergence had no access to sugar. Other mosquitoes were provided a 10% solution of corn syrup *ad lib*. All experiments were conducted on female mosquitoes only, given their role in malaria transmission and the importance of longevity to that role. Measurements were performed at various adult ages because water or energy storage strategies as well as stress resistance mechanisms change with age and may take trajectories that are population specific. All experiments were replicated three times on successive generations. Each replicate consisted of at least 20 mosquitoes per population, age group and acclimation regime. Total sample sizes are indicated for each experiment described below.

### Body mass, energy and hydration state

Body characteristics and energy reserves were determined on the day of emergence and at 2, 4, 6 and 8 days post-emergence (always ~10 am for convenience and repeatability). Mosquitoes of a given age were individually weighed on a microbalance (Mettler Toledo, OH, USA; acc. 0.2 μg), dried overnight at 70°C and reweighed. Lipid content per mosquito was obtained by reweighing the dry carcass after soaking it in hexane overnight on a shaker. Glycogen content was obtained on pools of five mosquitoes, following methods modified from Van Handel [22]. Briefly, dry carcasses were ground in 200 μL of 2% sodium sulfate, and 200 μL of methanol was added, followed by vortexing, centrifugation at 4000 rpm and removal of the supernatant. The pellet containing glycogen was redissolved in 1 mL water and 1/10^th ^was used for quantification (amount per half mosquito). Anthrone solution (750 mg anthrone in 530 mL 72% sulfuric acid) was then added to each tube up to 1.5 mL, tubes were heated at 90°C for 17 min, and glycogen was quantified spectrophotometrically at 625 nm. Overall, a total of 60 mosquitoes per age group and per population were analyzed. For glycogen, 40 additional mosquitoes were used to obtain a total sample size of 20 per age group and population.

### Desiccation resistance

Desiccation resistance (DR) of adult females at 0, 4 and 8 days post-emergence was compared using a method previously described in Gray and Bradley [18]. On a given day individual females were CO_2_-anesthetized, weighed, then placed in a glass vial sealed with a foam stopper, Drierite^® ^and Parafilm^® ^(RH < 10%). Experiments on emerging mosquitoes were initiated at 8 am and those on 4- and 8-day old mosquitoes were initiated at 12 am, for logistical purposes. The sealed vials were returned to the insectary and survival was assessed hourly until all mosquitoes were dead. A mosquito was declared dead when it could no longer fly or right itself. At this point it was reweighed and placed in the drying oven overnight. Dry mass was obtained the following day. A total of at least 60 females per age group and population was analyzed.

DR expressed in hours represents the time to death in a desiccating environment. Initial body water mass was determined by subtracting dry mass from initial body mass, and water mass at death by subtracting dry mass from body mass at death. Initial body water and body water at death were expressed relative to dry mass (*i.e*., divided by dry mass) to permit comparison between groups. Mass specific body water at death was used to describe dehydration tolerance, which represents the hydration level below which an animal cannot survive. Water loss rate (in μg.h^-1^) was calculated by subtracting water mass at death from initial water mass and dividing by DR.

### Acclimation effects on body mass, energy, hydration and desiccation resistance

On the day of emergence, replicate adult populations were placed in either the humid insectary or an insectary maintained at 60% RH and 30°C ("dry environment"), where they remained for eight days. The characteristics of eight day-old females reared in either the dry or humid environment were compared. The purpose was to investigate each population's ability to acclimate to a more stressful environment, albeit one almost certainly experienced by *A. gambiae *at the drier extremes of its geographic range [23]. At least 60 mosquitoes were tested for each population and each acclimation regime.

### Statistical analyses

The effects of desiccation and acclimation on body characteristics were analysed by linear factorial mixed-effects models under a nested design, with population (*i.e*., karyotype), acclimation, or age as fixed effects, and replicates, population within replicates, and acclimation or age within populations as random effects. For those analyses where the mosquito age was used as a covariable, the statistical significance of differences in water loss rate, initial water content and water content at death between populations (POP factor) in relation to age effects (AGE covariable) was assessed by generalized linear models (GLMs) with POP and AGE and their interaction nested within replicates (factor REPEAT). Given the approximately hyperbolic shape of the relationship in most cases, the best fitting GLM models, judged according to the Akaike Information Criterion (AIC), were those with an inverse link function, gamma errors, and the age covariable square root-transformed. Parametric survival regression was used to test the effect of population (2L+^a ^vs. 2L*a*), initial water content, water content at death, and water loss rate on survival under desiccation stress. Survivorship was modeled using the Weibull distribution. As all mosquitoes eventually died during the desiccation resistance test, survival times were not censored. All analyses were performed with the software R v.2.9.2  using the additional libraries *survival *[24], and *lme4 *[25].

## Results

### Body characteristics of 2La and 2L+^a ^populations

**Figure 1:**
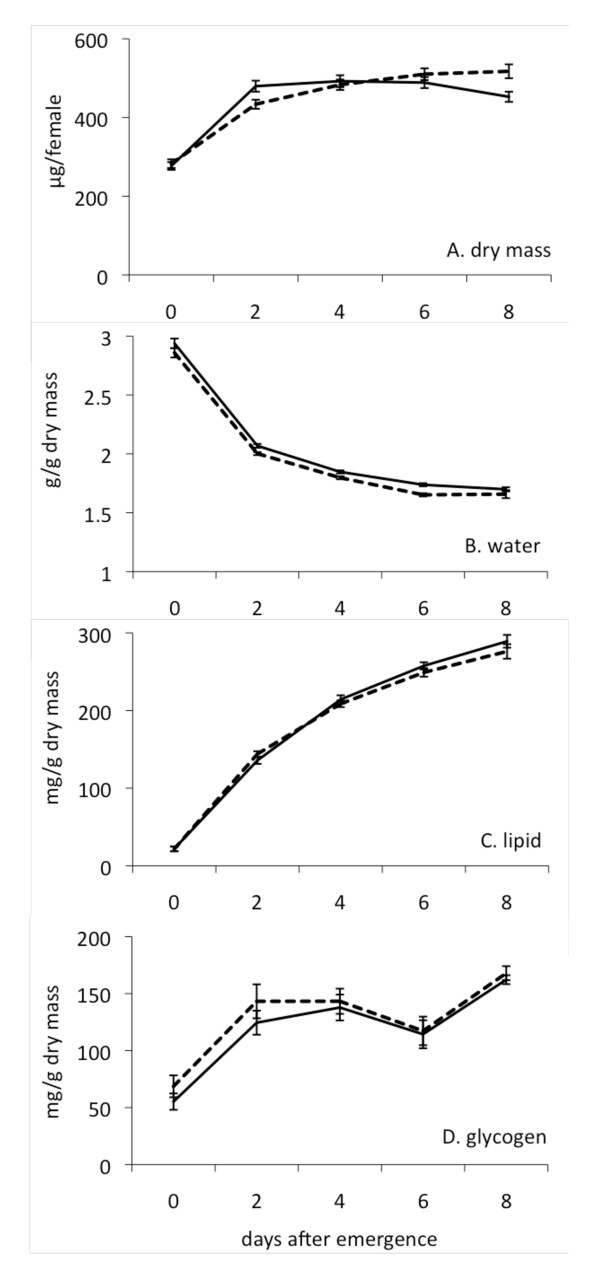
**Change in female *A. gambiae *body characteristics with age, by karyotype**. Solid lines represent 2L+^a ^females; broken lines represent 2La females. Bars represent S.E.

Figure 1 shows the change in dry mass (A), dry mass specific body water (B), lipid (C) and glycogen (D) in both populations from emergence to 8 days post-emergence. Overall there was no difference between populations in dry mass (*P *= 0.396), water content (*P *= 0.219), lipid (*P *= 0.641), or glycogen (*P *= 0.205). Dry mass strongly increased in the first 2 days while specific body water content dropped, then both remained stable at later ages. Lipid and glycogen both increased during the first week of adult life, although lipid increased progressively while glycogen stores were mostly boosted in the 2 days post-emergence.

### Desiccation resistance

**Figure 2:**
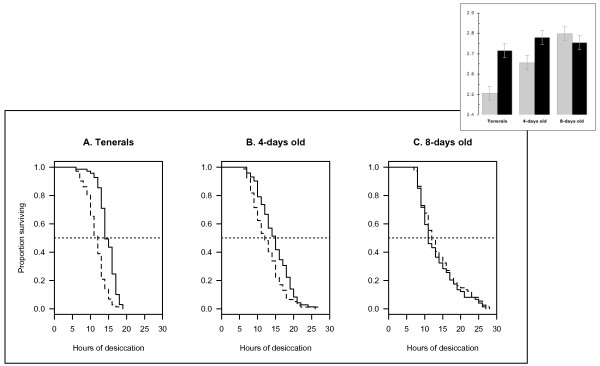
**Survivorship by karyotype (2La, continuous lines; 2L+^a^, dashed lines) of female *A. gambiae *at three age groups when submitted to a desiccation resistance test**. The insert shows the estimated value of the scale parameter (± standard error) of the Weibull hazard function for the six populations (black bars: 2La, light grey bars: 2L+^a^). Larger values of the scale parameter correspond to lower hazards and increased survival. Shape parameter of the full regression model = 0.291.

The survival of 2La females submitted to desiccation stress remained constant across the first two age groups tested, and then decreased (14.7, 14.7, and 13.3 hours for tenerals, 4-days old, and 8-days old mosquitoes, respectively). Conversely, the survival of 2L+^a ^females increased with age (11.6, 12.6, and 13.9 hours, respectively). This pattern, plotted in Figure 2, resulted in statistically significant differences in survival between the two karyotypes in the first two age groups (tenerals and 4-days old), with survival leveling off to the same average between the two populations in 8-days old mosquitoes (increase in deviance caused by removal of the POPULATION × AGE interaction term from the full regression model = 14.1; d.f = 2; *P *< 0.001; Figure 2).

**Figure 3:**
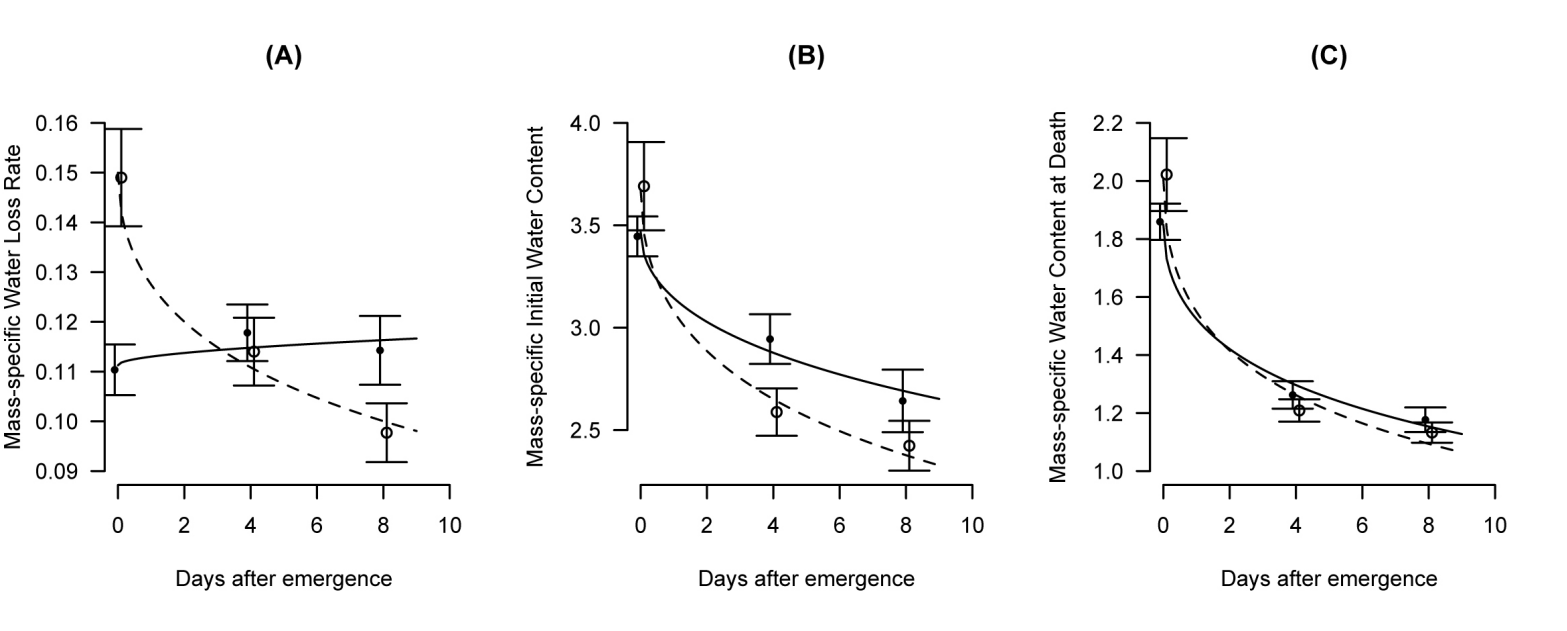
**Changes with age in mass-specific values of (A) water loss rate, (B) initial water content, and (C) water content at death by karyotype in female *A. gambiae *submitted to desiccation stress tests**. 2La, closed circles and continuous lines; 2L+^a^, open circles and dashed lines. Symbols and error bars represent means ± 95% confidence intervals. Lines depict the fitted generalized linear models. All values on the ordinate are in μg·μg^-1^. Water loss rates are expressed in μg·μg^-1^·hr^-1^.

To explore the effect of initial water content, water content at death, and water loss rate on survival under desiccation stress, these explanatory variables were fitted as covariates in a regression model containing the population (karyotype) and its interaction with each covariate as terms of the full model. All three explanatory variables had a highly significant effect on survival (*P *< 0.0001 in all cases). Table 1 shows that initial water content increased survival (positive regression coefficients), whereas water content at death and water loss rate decreased it (negative regression coefficients). The probability of death (the baseline hazard; intercepts in Table 1) was significantly different between karyotypes only in the case of the teneral and 4-day old age groups, confirming the result of the previous survival analysis (*cf *Figure 2). Table 1 also shows the statistical significance of the difference in the relative contribution to the hazard of water loss rate, initial water content and water content at death between the two populations (asterisks in Table 1). However, the effect of the explanatory covariates on karyotypes and age groups in Table 1 is confounded by body mass, given the highly significant correlation of dry mass with all three (initial water content: Pearson product-moment correlation coefficient r = 0.73, 95% confidence interval 0.68-0.77, d.f. = 436, *P *< 0.0001; water content at death: r = 0.71, 95% C.I. 0.66-0.75, d.f. = 436, *P *< 0.0001; water loss rate: r = 0.68, 95% C.I. 0.63-0.73, d.f. = 436, *P *< 0.0001). Accordingly, these variables were standardized with respect to dry mass, and their mass-specific values were plotted as a function of time after emergence (Figure 3). The statistical significance of differences between karyotypes in relation to age effects was assessed by generalized linear models (Table 2). Based on these analyses, the mass-specific water loss rate was significantly greater in 2L+^a ^compared to 2La tenerals. However, at 8 days post-emergence the pattern was reversed (Figure 3A), as indicated by the statistically significant interaction term in Table 2. Mass-specific initial water content was similar at emergence, but it diverged between 2L+^a ^and 2La karyotypes later (marginally non-significant interaction, Table 2), with 2La having proportionally more than 2L+^a ^(Figure 3B). Mass-specific water content at death significantly decreased with age in a similar pattern for both karyotypes (Figure 3C, non-significant interaction in Table 2). Thus, differences in survival between karyotypes were accounted for by lower water loss rates at emergence and increased water contents at 4 days in 2La individuals. By 8 days post-emergence, differences in survival might have disappeared because the larger initial water content in 2La karyotypes was counterbalanced by a higher water loss rate compared to 2L+^a ^karyotypes.

**Table 1:** Comparison of survival curves for 2L+^a ^and 2La karyotypes of *A. gambiae *females at different ages when submitted to a desiccation resistance test

**Parameter**	**Tenerals**			**4-days old**			**8-days old**		
						
	**2L+^a^**	**2La**		**2L+^a^**	**2La**		**2L+^a^**	**2La**	
Intercept	2.424	2.660***		2.460	2.698***		2.597	2.550	
Initial water content	0.0025	0.0024		0.0017	0.0014***		0.0014	0.0013	
Water content at death	-0.0028	-0.0022*		-0.0020	-0.0015**		-0.0012	-0.0011	
Water loss rate	-0.0227	-0.0360***		-0.0172	-0.0188*		-0.0213	-0.0172***	
Shape	0.0471			0.0707			0.0880		

Significant differences: * *P *< 0.05; ** *P *< 0.01; *** *P *< 0.001

Shown for the explanatory covariables are fitted regression coefficients of the scale parameter of Weibull hazard functions. Asterisks denote significant differences between pairs of coefficients from the two populations.

**Table 2:** Analysis of deviance for the effect of karyotype (POP) and days after emergence (AGE) on mass-specific water loss rate, initial water content, and water content at death during desiccation resistance tests of *A. gambiae *females.

**Response**	**Source**	**d.f**.	**Deviance**	**Residual** **d.f**.	**Residual** **Deviance**	**F**	***P***
Water loss rate	NULL	437	32.911				
	Repeat	3	1.147	434	31.764	5.22	0.104
	Repeat:POP	4	1.564	430	30.200	5.57	0.095
	Repeat:AGE	3	4.110	427	26.090	22.42	0.015
	Repeat:POP*AGE	3	2.616	424	23.474	15.75	0.024
							
Initial water content	NULL	437	29.115				
	Repeat	3	1.091	434	28.024	5.63	0.095
	Repeat:POP	4	0.299	430	27.724	1.16	0.470
	Repeat:sqrt(AGE)	3	11.582	427	16.143	102.12	0.002
	Repeat:POP*sqrt(AGE)	3	0.731	424	15.412	6.70	0.076
							
Water content at death	NULL	437	39.913				
	Repeat	3	2.718	434	37.195	10.57	0.042
	Repeat:POP	4	0.085	430	37.110	0.25	0.895
	Repeat:sqrt(AGE)	3	23.155	427	13.956	236.15	0.000
	Repeat:POP*sqrt(AGE)	3	0.365	424	13.590	3.80	0.151

### Effects of prior acclimation to dry conditions

**Figure 4:**
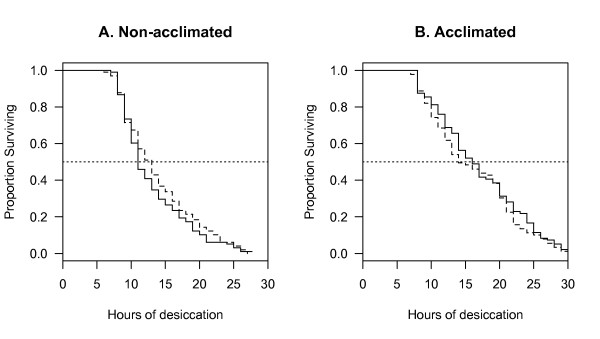
**Effect of prior acclimation on desiccation resistance of 8-day old *A. gambiae***. Survivorship curves of (A) non-acclimated and (B) acclimated females by karyotype (2La, continuous lines; 2L+^a^, dashed lines).

Prior acclimation significantly increased desiccation resistance of 8-day old *A. gambiae *(from 15.1 to 18.4 hrs on average, as estimated from survival regression; Figure 4). However, the response was similar for both karyotypes, resulting in no significant difference in survival between 2La and 2L+^a ^(Figure 4), as shown by the non-significant interaction between karyotype and acclimation factors in Table 3.

**Table 3:** Analysis of deviance of the effect of prior acclimation on the survival of 8-day old *A. gambiae *females in desiccation resistance tests.

**Source**	**d.f**.	**Deviance**	**Residual d.f**.	-2*LL	***P***
Null Model	NA	NA	379	2421.00	NA
Replicate	3	67.918	376	2353.08	<0.001
Replicate/Karyotype	4	3.631	372	2349.45	0.458
Replicate/Acclimation	4	64.646	368	2284.80	<0.001
Replicate/Karyotype * Acclimation	4	3.265	364	2281.54	0.515

LL: log-likelihood

Of the three explanatory variables, only standardized water loss rates significantly decreased in both populations following acclimation (Table 4, and Acclimation factor in Table 5). The difference between karyotypes in baseline water loss rate under normal, *i.e*. non-acclimated, conditions that was found in the previous desiccation resistance experiment for 8-day old mosquitoes was confirmed by this experiment (Karyotype factor in Table 5). The interaction between karyotype and acclimation, however, was not statistically significant, indicating that the response to acclimation was similar in both 2La and 2L+^a ^karyotypes (Table 5). A marginally non-significant difference between karyotypes in initial water content, regardless of acclimation, was found (Table 5); this is in agreement with the difference in initial water content between karyotypes at 8 days post-emergence found in the previous desiccation resistance experiment.

**Table 4:** Effect of prior acclimation on mass-specific responses (means ± SEM) by alternative karyotypes (2L+^a ^and 2La) in desiccation resistance tests of *A. gambiae *females.

**Response**	**Non-acclimated**		**Acclimated**	
	**2L+^a^**	**2La**	**2L+^a^**	**2La**
Water loss rate	0.099 ± 0.002	0.113 ± 0.003	0.071 ± 0.002	0.092 ± 0.002
Initial water content	2.43 ± 0.05	2.61 ± 0.06	2.20 ± 0.05	2.65 ± 0.06
Water content at death	1.13 ± 0.01	1.18 ± 0.02	1.08 ± 0.02	1.14 ± 0.02

**Table 5:** ANOVA results describing the efffect of prior acclimation on mass-specific responses by *A. gambiae *females to desiccation resistance tests.

**Response**	**d.f**.	**Sum Sq**.	**Mean Sq**.	**F**	***P***	
**A. Water loss rate**						
Karyotype	1	0.0098	0.0098	18.34	0.013	*
Acclimation	1	0.0292	0.0292	54.49	<0.001	***
Karyotype * Acclimation	1	0.0004	0.0004	0.80	0.385	
**B. Initial water content**						
Karyotype	1	1.2044	1.2044	5.48	0.079	
Acclimation	1	0.0874	0.0874	0.40	0.545	
Karyotype * Acclimation	1	0.2262	0.2262	1.03	0.325	
**C. Water content at death**						
Karyotype	1	0.09704	0.09704	3.79	0.123	
Acclimation	1	0.05002	0.05002	1.95	0.200	
Karyotype * Acclimation	1	0.00031	0.00031	0.01	0.922	

In terms of body characteristics, only mass-specific lipid and glycogen content were significantly affected by acclimation (Tables 6, 7). In both cases, the response differed according to karyotype (significant interaction terms in Table 7): 2La karyotypes decreased lipids but increased glycogen content; 2L+^a ^karyotypes increased lipids but decreased glycogen content following acclimation (Table 6). Differences in dry mass and standardized water content were not statistically significant (Table 7), although the larger average water content of 2La vs. 2L+^a ^karyotypes was consistent with results from the previous desiccation resistance experiment.

**Table 6:** Effect of prior acclimation on body characteristics (means ± SEM) of alternative karyotypes (2L+^a ^and 2La) in desiccation resistance tests of *A. gambiae *females

**Response**	**Non-acclimated**		**Acclimated**	
	
	**2L+/+**	**2L*a*/*a***	**2L+/+**	**2L*a*/*a***
Dry mass (μg)	610 ± 18	632 ± 19	664 ± 15	646 ± 15
Water content^1 ^(μg·μg^-1^)	1.621 ± 0.016	1.673 ± 0.014	1.571 ± 0.015	1.669 ± 0.016
Lipid content^1 ^(μg·μg^-1^)	0.295 ± 0.005	0.278 ± 0.004	0.333 ± 0.005	0.268 ± 0.004
Glycogen content^1 ^(μg·μg^-1^)	0.156 ± 0.006	0.159 ± 0.006	0.146 ± 0.005	0.167 ± 0.006

^1^Standardized by dry mass

**Table 7:** ANOVA results describing the effect of prior acclimation on body characteristics of alternative karyotypes (2L+^a ^and 2La) in desiccation resistance tests of *A. gambiae *females.

**Response**	**d.f**.	**Sum Sq**.	**Mean Sq**.	**F**	***P***	
**A. Dry Mass**						
Karyotype	1	168	168	0.01	0.925	
Acclimation	1	28230	28230	2.17	0.179	
Karyotype * Acclimation	1	6467	6467	0.50	0.490	
						
**B. Water Content^1^**						
Karyotype	1	0.0469	0.0469	2.40	0.196	
Acclimation	1	0.0051	0.0051	0.26	0.624	
Karyotype * Acclimation	1	0.0105	0.0105	0.54	0.473	
						
**C. Lipid Content^1^**						
Karyotype	1	0.0316	0.0316	18.50	0.013	*
Acclimation	1	0.0043	0.0043	2.53	0.150	
Karyotype * Acclimation	1	0.0161	0.0161	9.43	0.007	**
						
**D. Glycogen Content^1^**						
Karyotype	1	0.00288	0.00288	7.72	0.050	*
Acclimation	1	0.00006	0.00006	0.17	0.694	
Karyotype * Acclimation	1	0.00170	0.00170	4.56	0.049	*

^1^Standardized by dry mass

## Discussion

This study sought to establish whether the 2La inversion in *A. gambiae *confers a physiological advantage under arid conditions. Indeed, it was demonstrated that young adult females carrying the 2La inversion resist desiccation longer than their 2L+^a ^counterparts. Furthermore this difference in DR is age specific: the difference between populations was most pronounced on the day of emergence and still present in four day-old adults, but absent by eight days. The first few days of adult life include flying away from the emergence site, maturation of multiple organs, cuticle hardening, swarming, mating and orienting towards suitable feeding areas [26]. The first day of adult life may be particularly stressful as any fluid losses occurring can only be countered after dusk, when young mosquitoes begin seeking for nectar sources [27]. These activities subject mosquitoes to stressful and potentially lethal environments; the difference in DR may confer a competitive advantage to the 2La karyotype that could help explain its high frequency in arid habitats.

DR is determined by initial and final body water contents as well as the rate of body water loss. The measurement of all three parameters revealed that emerging females of either karyotype differed most strongly in their rate of water loss. Specifically, teneral 2La females had a significantly lower water loss rate than their 2L+^a ^counterparts. In fact, when comparing age groups, 2La females had a similar rate of water loss at emergence and later ages, whereas 2L+^a ^females experienced their highest rate of water loss at emergence.

Variation in water loss rate most likely involves modifications in the physical characteristics of the main barrier to water loss, the cuticle and/or its waxy surface. Although water loss can also occur via the spiracles during respiration, it is thought that respiratory water loss contributes very little to overall water loss in insects [28-31]. Diuresis also contributes to water loss. *A. gambiae *discharge excess fluids during the first 5 min post-eclosion to facilitate flight [32]. In addition, diuresis follows feeding and is thought to be stimulated by abdominal distension [26]. However, it is unlikely that the observed differences in water loss rate were due to diuresis after feeding, as mosquitoes had no access to liquids during the desiccation bout.

### Modulating cuticular water loss

The cuticle may be the principal source of dehydration and the one that, if modified, can most promote water conservation. Cuticular water loss can be reduced by variation in the quantitative or qualitative production of cuticular hydrocarbons, which are the main components of the waxy layer covering the epicuticle. Beetles, scorpions and other arthropods living in desert environments manage to reduce cuticular permeability by producing more waxes than animals found in mesic environments [33,34]. *Culex pipiens *mosquitoes also increase the amount of cuticular lipids in preparation for winter diapause [17]. The chain length of cuticular hydrocarbons, which correlates positively with melting point temperature, may also influence the waterproofing qualities of the waxy layer by affecting its stability at high ambient temperatures [35]. Cuticular hydrocarbon analyses of *A. arabiensis *and two molecular forms of *A. gambiae *collected from Burkina Faso uncovered no qualitative differences in chain length within or among groups, but quantitative differences in hydrocarbon abundance were noted [36]. It is possible that these differences reflect local climatic conditions, but they are probably not associated with alternative arrangements of the 2La inversion, which is fixed in *A. arabiensis *and similarly fixed or nearly so in *A. gambiae *populations from the dry savanna of Burkina Faso, where most specimens were collected.

Another possible mechanism to modulate cuticular water loss may involve cuticular proteins. Until now these proteins, which are structurally important components of the cuticle, have never been implicated in cuticular waterproofing. Yet recent work suggests that cuticular protein genes can be up-regulated by environmental stress, including desiccation [37]. Interestingly, a chromosomal region within inversion 2La that is implicated in its maintenance [12] contains a cluster of 39 genes encoding cuticular proteins of the RR-2 consensus, a major family of structural components of the hard cuticle [38]. Following an appropriate regimen of stress, differences between populations in the expression profiles of gene(s) encoding these cuticular proteins could be suggestive of their involvement in cuticular permeability.

### The influence of body water content on DR

Although body water content was not consistently different between populations, it is apparent from the desiccation results for four day-old mosquitoes that this trait can have significant effects on DR (Figure 3). Body water varies throughout the day; it is lost by transpiration, respiration and excretion and is replenished by fluid absorption during feeding. Measurements of body characteristics including body water content (Figure 1) were performed at 10 am, whereas mass measurements prior to the desiccation assays were performed before 8 am for teneral mosquitoes and at 12 am (midnight) for 4 and 8 day-old mosquitoes. As these measurement times were consistent between replicates, they allow for preliminary comparisons of daily variation in body water stores between 2La and 2L+^a ^populations. Whereas both populations had the same body water content at 10 am, water content differed between populations at the other time points, hinting at the possibility that alternative karyotypes may not manage their body water stores in the same way throughout the 24 h period. *A. gambiae *are nocturnally active and quiescent during the day; it is possible that differences in the timing (or frequency) of feeding (drinking) before entering daily quiescence may lead to differential survival of alternate karyotypes. Testing of this hypothesis will require investigating the diurnal pattern of sugar (or nectar) feeding in both populations.

### Acclimation effects on DR and energy stores

Adult females of both populations were reared in an environment mimicking more arid conditions (both warmer and drier) to test the hypothesis that 2La females were better equipped than their 2L+^a ^counterparts to respond to hygrically stressful environments. In fact, both groups effectively acclimated to this environment by reducing their rate of water loss, suggesting a phenotypic plasticity in water loss rate that is not associated with alternative arrangements of the 2La inversion. Furthermore, both groups increased their body size, a change leading to decreased ratio of surface area to volume, which is advantageous in dry environments as it reduces the mass specific rate of water loss [39,40]. On the other hand, changes in other body characteristics following acclimation suggest karyotype-specific effects on energy stores that may affect stress resistance. Glycogen is thought to enhance DR by increasing water storage, as it binds up to 5 times its weight in water [39,41]. Glycogen stores increase in response to selection for DR in *D. melanogaster *[42] and both are significantly correlated among several populations [43]. Lipid storage correlates with starvation resistance [44], another possible adaptation to aridity. These traits did not differ among females of alternative karyotypes in the absence of acclimation. However, the populations responded differently to acclimation: 2L+^a ^females boosted their lipid stores and decreased glycogen content while 2La females decreased lipids and increased glycogen. This result could reflect an inversion-associated difference in life history strategy. Lipid is an efficient form of energy storage as for the same mass of stored product, lipid yields 10 times more energy than glycogen (particularly since glycogen is stored with water) [39]. Lipid stores are boosted in some female mosquitoes in preparation for winter diapause [45,46]. A similar strategy has been observed for various animals in preparation for aestivation during the hot dry season [47]. Evidence of true aestivation by *A. gambiae *is not compelling [48]. However, the different lipid storage response to acclimation by 2La versus 2L+^a ^karyotypes under controlled laboratory conditions and its implications for fecundity, immunity, longevity and other processes merits further investigation.

## Conclusion

This study is the first to link specific physiological traits with an inversion in the malaria vector *A. gambiae*, highlighting the fact that the 2La inversion can affect its carriers in multiple ways. It was demonstrated that the 2La inversion is associated with enhanced DR resulting from depression of water loss rate in young adult females, a trait that may contribute to the success of *A. gambiae *in arid regions. A possible difference in an environmentally induced life history strategy associated with alternative arrangements of the inversion also was uncovered. When reared in a dry environment, 2L+^a ^females boosted their lipid stores while 2La females elevated their glycogen content. Confirming these physiological differences in natural populations and determining their fitness implications are important next steps. Polymorphism for the 2La inversion creates heterogeneity in the stress response within *A. gambiae*, which could directly or indirectly reduce the efficacy of vector control measures, and influence the reaction of vector populations to environmental variation including climate change. The longterm-goal of understanding the functional significance of inversion polymorphism generally, and the 2La inversion polymorphism in particular, will be realized by the integration of physiological, behavioral, and molecular information that will shed light on the adaptive mechanisms that have made *A. gambiae *so ecologically successful.

## Competing interests

The authors declare that they have no competing interests.

## Authors' contributions

EG and NB conceived the study. EG and KR performed the experiments. EG and CC analyzed results. EG wrote the manuscript, NB and CC helped revise it. All authors read and approved the final manuscript.
